# Protein Ensemble Generation Through Variational Autoencoder Latent Space Sampling

**DOI:** 10.1021/acs.jctc.3c01057

**Published:** 2024-03-28

**Authors:** Sanaa Mansoor, Minkyung Baek, Hahnbeom Park, Gyu Rie Lee, David Baker

**Affiliations:** † Department of Biochemistry, University of Washington, Seattle, Washington 98195, United States; ‡ Institute for Protein Design, University of Washington, Seattle, Washington 98195, United States; § Molecular Engineering Graduate Program, University of Washington, Seattle, Washington 98195, United States; ∥ School of Biological Sciences, Seoul National University, Seoul 08826, Republic of Korea; ⊥ Brain Science Institute, Korea Institute of Science and Technology, Seoul 02792, Republic of Korea; # Howard Hughes Medical Institute, University of Washington, Seattle, Washington 98195, United States

## Abstract

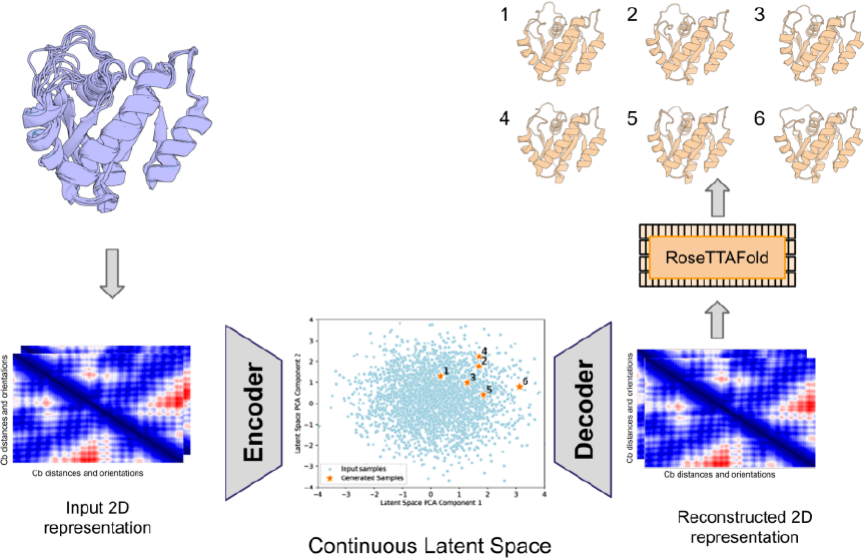

Mapping the ensemble of protein conformations that contribute to function and can be targeted by small molecule drugs remains an outstanding challenge. Here, we explore the use of variational autoencoders for reducing the challenge of dimensionality in the protein structure ensemble generation problem. We convert high-dimensional protein structural data into a continuous, low-dimensional representation, carry out a search in this space guided by a structure quality metric, and then use RoseTTAFold guided by the sampled structural information to generate 3D structures. We use this approach to generate ensembles for the cancer relevant protein K-Ras, train the VAE on a subset of the available K-Ras crystal structures and MD simulation snapshots, and assess the extent of sampling close to crystal structures withheld from training. We find that our latent space sampling procedure rapidly generates ensembles with high structural quality and is able to sample within 1 Å of held-out crystal structures, with a consistency higher than that of MD simulation or AlphaFold2 prediction. The sampled structures sufficiently recapitulate the cryptic pockets in the held-out K-Ras structures to allow for small molecule docking.

A major challenge in drug discovery is identifying cryptic binding pockets that can be targeted by small molecule drugs.^1−3^ Despite considerable advances in single-state native protein structure prediction with AlphaFold^4^ and RoseTTAFold^5^ in the past several years, generating plausible ensembles of structures that can be populated upon binding a small molecule or during protein function remains an outstanding problem – AlphaFold and RoseTTAFold generate single structures rather than ensembles. Molecular dynamics (MD) trajectories generate protein ensembles by simulating protein motion around the native structure and are often used to generate ensembles prior to small molecule docking calculations but often fail to identify cryptic ligand binding pockets not present in the unbound structure^1−3,6^ or require very long and hence highly compute-intensive simulations (typically subto-several microsecond level).^7−9^ Rosetta fragment assembly and minimization^10^ and kinematic closure^11^ methods have been used to model protein and loop conformational diversity, but these methods have typically not sampled the types of conformational changes involved in cryptic pocket formation. On the deep learning side, variational autoencoders, which project complex data into a smaller dimension latent space, have been used to generate alternative backbones for general protein design tasks such as de novo design of 64 residue backbones,^12^ graph-based protein design,^13^ and Ig-fold modeling.^14^ VAEs have been used previously to sample the conformational space of proteins but have required visual inspection of the trained latent space to sample^15^ or have focused on mapping correlative fluctuations in extensive MD simulations of both the apo and holo states of a target protein.^16^

We reasoned that sampling within the latent space of variational autoencoders could provide a solution to the ensemble generation problem for a specific protein sequence. Unlike most previous VAE approaches, which have been trained on many different proteins, the challenge of a protein specific VAE is that there is limited training data. We reasoned that this limitation could be overcome by supplementing available crystal structures of the protein of interest in alternative conformations with snapshots from short MD trajectories started from each of these structures. For exploring this approach, we chose the critical cancer target K-Ras as a model system due to its considerable therapeutic importance and the many available structures.^17^

We began by exploring different VAE architectures, training on ensembles of MD simulations from alternate crystal forms of K-Ras (full details in the Methods section), and evaluating the quality of 3D reconstruction following encoding and decoding. For encoding 3D structural information, we chose to use the 2D RoseTTAFold template features; the VAE training seeks to minimize the difference between input and output features for each training set structure. The reconstructed template features are then used as input template features for 3D structure generation with RoseTTAFold, along with the amino acid sequence. We evaluate the accuracy of reconstruction by computing the RMSD between the input and output atomic coordinates (computed over C-alpha atoms here and throughout the manuscript). We generated new samples by guided exploration in the latent space, followed by 3D coordinate generation with RF (RoseTTAFold).

The reconstruction accuracy of crystal structures not included in the training set provides a rough lower bound on the accuracy with which our approach can recapitulate conformations of interest. For each available K-Ras structure, we trained a VAE leaving out this structure and others within 1 Å coordinate RMSD and evaluated the accuracy of reconstruction following RoseTTAFold^5^ 3D coordinate generation. We obtained the best results with the soft-introspective VAE architecture (Figure S1), and the accuracy of reconstruction plateaued at ∼256 latent space dimensions (Figure S2). For most of the targets (13/20), the reconstruction was within 1 Å coordinate RMSD of the input structure; for comparison, only 2/20 AF2 models were of subangstrom accuracy (Figure 1 and Table S1).

**Figure 1:**
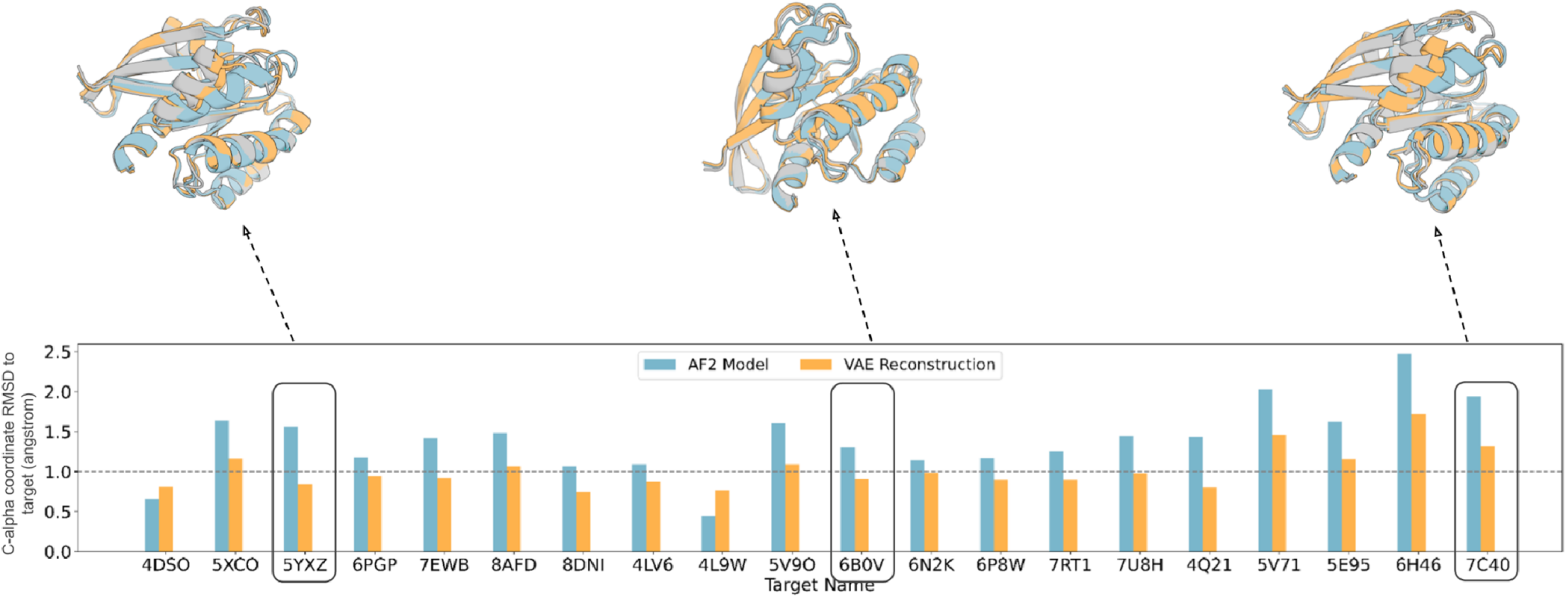
VAE structure reconstruction accuracy. C-alpha coordinate RMSD (angstrom) of the closest AF2 predicted model and the reconstructed model from the VAE decoded template features generated using RoseTTAFold. Structural superimpositions for 3 targets are highlighted on the top with the target crystal in gray, the AF2 prediction in blue, and the VAE reconstruction in orange.

We next explored the possibility of generating plausible K-Ras ensembles by sampling in the latent space of trained VAEs. To help ensure that the sampled structures remained broadly consistent with the sequence and were physically plausible, we guided sampling by the consistency with the AF2 predicted distance distribution for the amino acid sequence. Samples were generated from a normal distribution with a mean of 0 and variance of 1, decoded into the corresponding C-beta (Cb) distance map, the categorical cross-entropy (CCE) to the AF2 predicted distogram for the sequence was computed, and local optimization in the latent space was carried out through gradient descent on the CCE value, limiting the total (latent space) distance traversed from the starting point to prevent convergence. Principal component analysis (PCA) on the latent space (Figure 2) showed that the generated and training samples have a similar distribution and surround the target crystal structure as intended.

**Figure 2:**
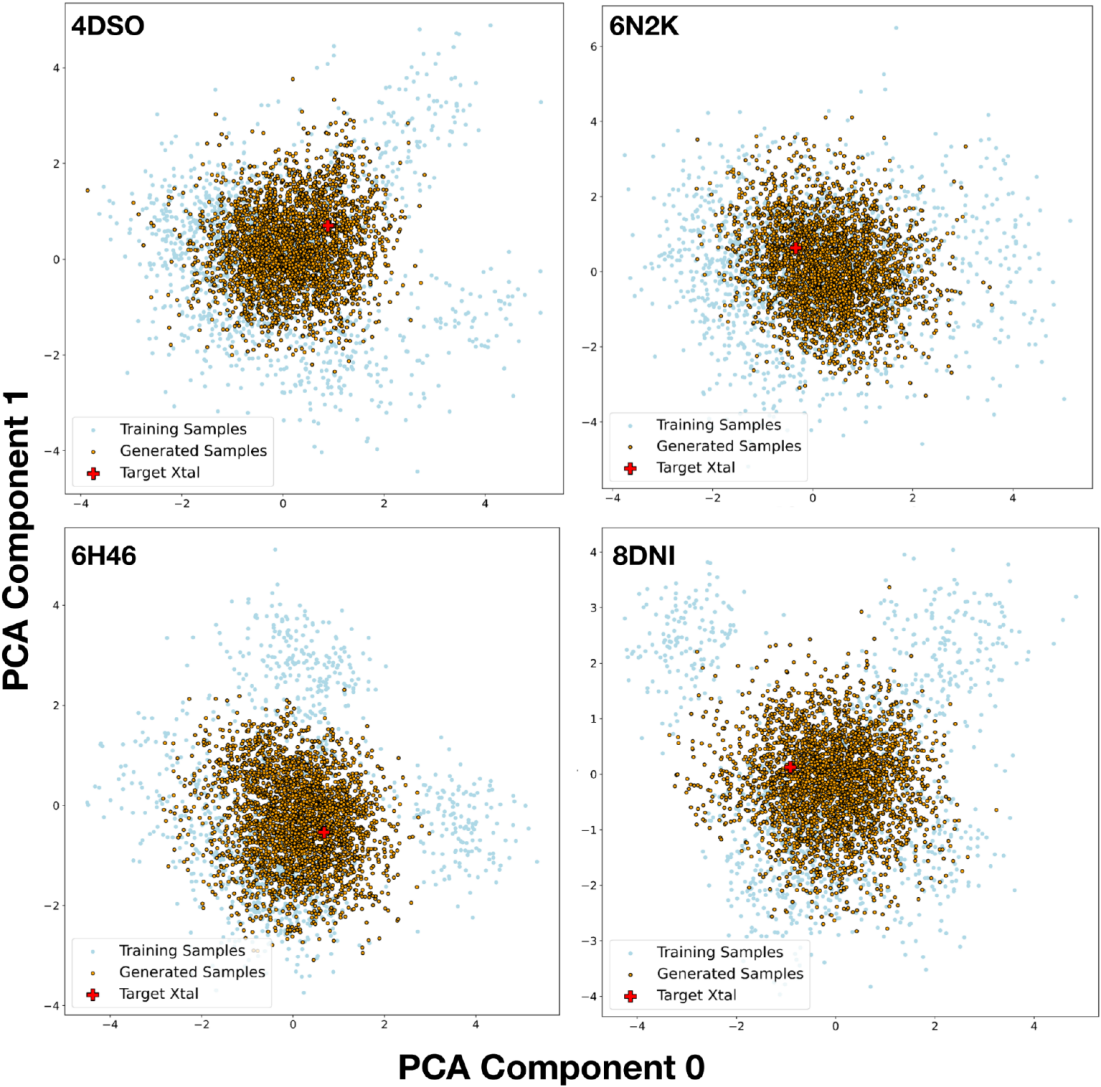
Latent space PCA analysis. Each subplot displays a 2D PCA projection of the 256-dimensional latent space. The training and generated samples have similar distributions and surround the crystal structure.

Using this VAE guided sampling approach, we generated K-Ras structure ensembles for each target structure, again holding out the target, MD simulations starting from the target, along with all crystal structures within 1 Å coordinate RMSD of the target, and MD snapshots derived from them. Following decoding and RF structure generation, the coordinate RMSD to the target crystal was computed over either the entire structure or just the ligand binding pocket (residues with side chain atoms within 5 Å of ligand atoms). An advantage of our approach is that ensembles can be generated quite rapidly (compared to MD simulations, for example), and as expected, the closest RMSD to the held-out structures decreases with increasing number of samples (Figure S3). For comparison, we provided the template features of the training set MD simulation snapshots as direct input to RoseTTAFold (MD + RoseTTAFold). We found that ensembles of 3000 generated structures sampled more closely to the held-out crystal structures than the closest training set crystal structure, the closest training set MD simulation snapshot, the closest MD + RoseTTAFold structure, and the closest AF2 model for most targets (Figure 3 and Table S2; the variation in the input training crystals impacts the closeness of the generated structures to the target crystal; Figures S4 and S5A,B). The comparison with AF2 is vital as it showcases the current state-of-the-art in single-state structure prediction; AF2 generates diverse structures for each target by incorporating variations in input structural templates and input MSA features (Table S5, ″1.12.1 Training procedure,″ in the Supporting Information, Jumper et al., 2021^4^), thereby providing valuable insights into protein conformational diversity.

**Figure 3:**
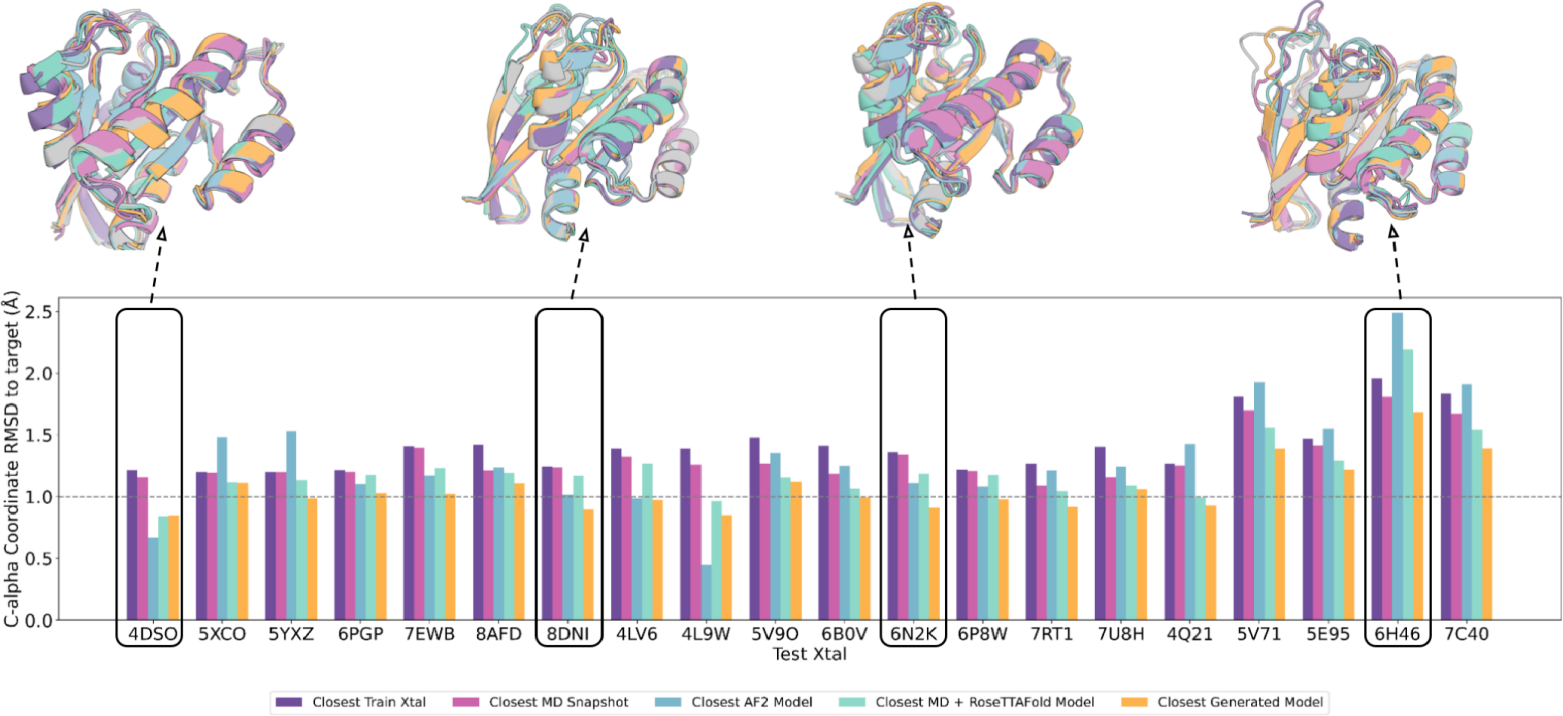
K-Ras overall structure reconstruction evaluation. The VAE enables sampling closer to held-out K-Ras crystal structures than MD, MD template features passed into RoseTTAFold, or AlphaFold generated structures. For each test crystal structure (name below bars), a VAE model was trained using MD simulation data from all crystal structures with greater than 1 Å C-alpha RMSD and used to generate a structure ensemble. Bars indicate the coordinate error to the test crystal of the closest train crystal, the closest training sample, the closest AF2 model, the closest MD + RoseTTAFold sample, and the closest VAE generated sample.

For small molecule docking calculations, sampling of alternative ligand binding pocket geometries is particularly important. Comparison of the C-alpha RMSD over the ligand binding pocket residues between the closest sampled conformation in the generated ensembles and the held-out structures showed that the ensembles sample closer than the closest training MD snapshot or crystal structure in most cases (Figure 4). Structural superimpositions show that the generated samples do not clash with the superimposed ligand from the target structure, highlighted in orange, and therefore can be docked without hindrance, whereas for the closest train crystal and the closest AF2 model, there are significant clashes (Figure 4 and Table S3).

**Figure 4:**
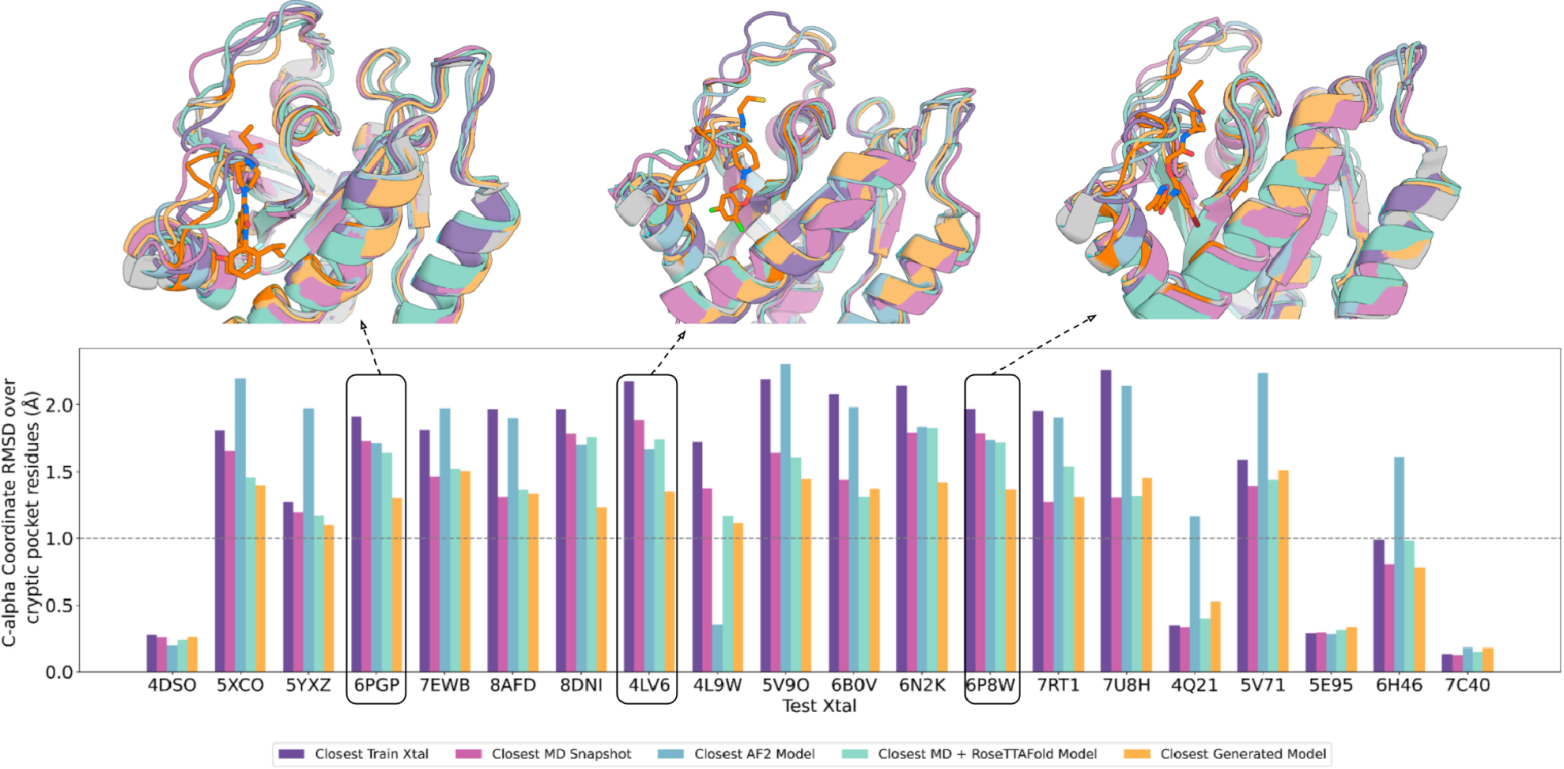
K-Ras cryptic pocket reconstruction evaluation. As in Figure 3, but with the C-alpha coordinate error to the test crystal structure computed over only the binding site residues (defined as the residues within 5 Å in C-alpha coordinate space of the ligand binding pocket). The structural superimpositions (top) show the ligand inhibitor docked in the target crystal with the cryptic binding pocket and the ligand highlighted in orange on the target crystal structure.

We used the physically based Rosetta GA-ligand docking method to dock ligands onto all the models generated from the VAE, the training examples, and the AF2 models. Consistent with the above observations, the RMSD over the ligand atoms was consistently lower for the ensemble generated samples than that for the AF2 predictions and lower in most cases than the docks to the MD ensembles (Figure 5 and Tables S4 and S5). While consistent, the improvements were relatively small; an improved ligand docking method could benefit more from better modeling of the binding pocket, particularly for larger bound partners, such as 6H46 with a DARPIN peptide and 5E95 with the NS1 synthetic binding protein.

**Figure 5:**
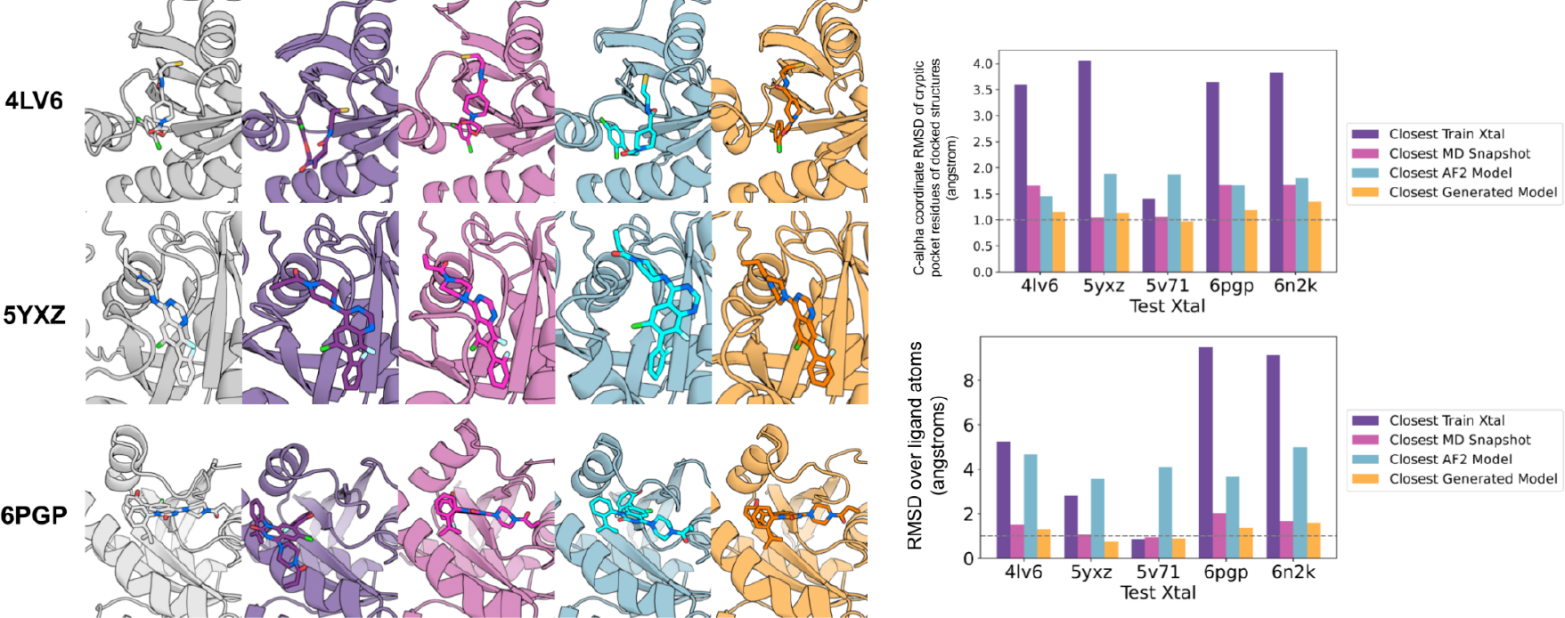
Small molecule docking into VAE generated ensembles. Ligands from held-out crystal structures were docked into protein conformers using the GA-ligand dock. Left: the held-out crystal structure complex (column 1) and the closest docked complex (in terms of coordinate RMSD over the ligand atoms) among the training set crystal structures (column 2), the MD snapshots (column 3), the AlphaFold models (column 4), and the VAE ensembles (column 5). The closest C-alpha RMSDs of the cryptic pocket of docked structures and lowest RMSD over ligand atoms (ligand RMSD) are indicated on the bar charts on the right.

## Discussion

Our VAE-based sampling approach enables extrapolation from combinations of MD simulation snapshots initiated from multiple known crystal structures to generate ensembles of conformers that more closely resemble held-out crystal structures. These ensembles can be generated with low computational cost (compared to the input trajectories) and sample alternative ligand binding site geometries for small molecule ligand docking. We go beyond previous studies using VAEs to model the space sampled by MD simulations by taking advantage of the sophisticated understanding of protein sequence–structure relationships implicit in the AF2 and RF deep neural networks in two ways: first, we use the AF2 predicted distance distributions to focus the latent space sampling on regions consistent with the amino acid sequence, and second, we use RF to generate 3D coordinates from the output distance maps, which ensures physical realism and local sequence-structure compatibility.

There are clear paths forward for improving our approach. First, the reconstruction error of ∼1 Å C-alpha coordinate RMSD for the known crystal structures is reasonable, but the challenge is that the differences between many of the different conformations are also of this order, limiting the ability of our approach to precisely sample alternative states. VAE architectures with lower reconstruction errors could likely improve the method as could training the VAE on the FAPE loss following RoseTTAfold coordinate generation (we did not observe this in preliminary tests, but this warrants further exploration). Second, while the AF2 CCE metric provides a reasonable guidepost, AF2 is trained to generate single structures, and hence, the use of this measure to guide sampling could limit diversity. Better results could perhaps be obtained by minimizing toward a predicted ensemble of structures for a given target or subsampling the target MSA during RoseTTAFold structure generation^18^ to introduce more diversity in output structures. Despite these limitations, our results show the utility of generative models for modeling the conformational ensembles that determine protein function and drugability.

## Methods

### Input Data Setup and Incremental Learning

For the input data set, we began by selecting distinct K-Ras conformations deposited in the PDB that are at least an angstrom (calculated over C-alpha coordinates) away from each other as our ‘training set crystal structures.’ In addition to the RMSD cutoff filter, we also selected conformations that had a deposited/known inhibitor. We selected 20 K-Ras structures with these criteria. We ran MD simulations for 10 ns starting with each K-Ras crystal structure in the ligand-free conformation (apo) and selected every 50 ps snapshot from 5 independent trajectories, giving a total of 1000 MD snapshots for each starting structure. AMBER19SB force field^19^ with TIP3P water model^20^ was used in a periodic boundary box. Langevin dynamics was run at a constant temperature of 300 K and pressure of 1 atm. For each target crystal, the training data consisted of MD snapshots of the training set crystal structures that were at least an angstrom (calculated over C-alpha coordinates) away from it. The final 20 K-Ras conformations that we chose were 4DSO, 5XCO, 5YXZ, 6PGP, 7EWB, 8AFD, 8DNI, 4LV6, 4L9W, 5V9O, 6B0V, 6N2K, 6P8W, 7RT1, 7U8H, 4Q21, 5V71, 5E95, 6H46, and 7C40. All 3D structures were converted to 2D template features from RoseTTAFold.^5^ The, 2D template features take the form of a tensor capturing 6D transformations between every pair of residues within a 20 Å range, specifically focusing on Cβ–Cβ distances. These features are extracted from the Cartesian coordinates of the N, Ca, C, and Cb atoms. The 6D coordinates encompass pairwise distances and angles (omega, theta, and phi). We chose to use the raw distance and orientation values for training the model for a more interpretable latent space.

After the first round of training using only MD snapshots as the training data, we then generated 3000 samples from the latent space that were optimized for the score metric and passed the diversity filter (following the protocol laid out in the Sampling in Latent Space Through Gradient Optimization of Score Metric (CCE) section). These 3000 generated structures were then concatenated on the initial MD snapshot training set to form an ‘incremental learning’ training set of structures for the model. Using this new set, for each target, the training runs were set up again from scratch. Incremental learning in this case benefits the VAE by providing a larger and more diverse set of structures for exploration, improving the representation of structural diversity, refining metric optimization, and ultimately increasing the accuracy of the generated samples to the target crystal.

### Soft Introspective VAE Objective and Training

We found best results using a Soft-Introspective VAE architecture,^21^ which has been shown to have higher output resolution than the vanilla VAE.^22^ The objective function of this model, along with the traditional VAE objective function of reconstruction loss and KL divergence, has adversarial losses incorporated like GANs^23^ but is trained introspectively. In the case of SI-VAEs, the encoder is the implicit ‘discriminator’ where it is induced to distinguish, through the ELBO (evidence lower bound)^20^ values that it assigns to the real and generated samples. The decoder is the ‘generator’ where it is induced to generate samples to ‘fool’ the encoder (discriminator). However, unlike GANs, the SI-VAE model does not converge to the data distribution, but to an entropy-regularized version of it.^21^

Using default parameters from Daniel et al. (2020),^21^ encoder was trained with the following objective (eq 1):
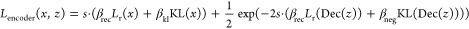
1where *L*_r_(*x*) = reconstruction loss, *s =* 2, β_rec_ = 10, β_kl_ = 1 × 10^–3^, β_neg_ = latent dimension = 256, and Dec = trained decoder of soft-introspective VAE.

The decoder was optimized using the following objective (eq 2):
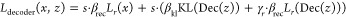
2where *L*_r_(*x*) = reconstruction loss, *s =* 2, β_rec_ = 10, β_kl_ = 1 × 10^–3^, and γ_r_ = 1.0

The reconstruction loss was the mean-squared error loss over all distances and orientations on the decoded template features from the model.

The VAE architecture comprises 3 ResNet blocks in both the encoder and decoder, with each block having 64 features. The encoder incorporates convolutional layers with batch normalization and leaky ReLU activation, followed by linear layers, leading to a latent space of 256 dimensions. The decoder consists of linear layers to reconstruct the input features followed by transposed convolutions and ResNet blocks. Leaky ReLU activation is applied throughout the network. Skip connections are implemented by using residual connections in the ResNet blocks. Batch normalization is used in both the encoder and decoder, with weight decay applied to prevent overfitting. Transposed convolutions handle upsampling in the decoder, and downsampling is achieved through convolutional layers with a stride of 2 in the encoder. This comprehensive architecture ensures effective encoding and decoding for VAE, contributing to its overall performance and reproducibility. The model was optimized using individual optimizers for the encoder and decoder, both of which were initialized with Adam (β1 = 0.9, β2 = 0.999) with learning rate 1 × 10^–3^, with an effective batch size of 64.

### Sampling in Latent Space Through Gradient Optimization of Score Metric (CCE)

To obtain the optimized structures using the trained decoder, we used gradient optimization in the latent space. We first randomly sample *n* numbers from the standard Gaussian distribution (mean = 0, standard deviation = 1) with dimensions equal to that of the latent space. The initialized latent space coordinates are set to be trainable. Each sample is then decoded into its respective template features, and Cb distances are discretized through radial basis function to ensure back-propagation. The score metric we chose to optimize is the minimum categorical cross-entropy (CCE) among all 5 AF2 predicted Cb distograms of the target structure and the generated Cb distances (eq 3). The Adam optimizer modifies the latent space sample to minimize this score metric. This process is repeated until convergence. To ensure that diversity is maintained, the latent space coordinates are restricted to explore only *d* (=10) euclidean distance in the latent space from their initial starting coordinates. The overall goal of this exploration technique is to search the latent space to find a better solution near the initial randomly generated coordinates. The final, converged latent space coordinates are decoded into their respective template features and passed into RoseTTAFold, along with the target MSA for structural modeling.
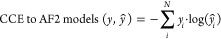
3where *N* is the number of categories in the predicted Cb distograms, *y*_*i*_ is the true distribution of Cb distances for target,  is the generated Cb distances.

### Docking Protocol

For each docking case, the inhibitor ligand was docked to the receptor model using the protein–ligand docking method Rosetta GALigandDock.^23^ The ligand atomic coordinates found in complex crystal structures were extracted and used to prepare the complex for ligand docking. The ligands were protonated and the AM1-BCC partial charges were calculated using the tools provided by openbabel, Antechamber in the AMBER suite, and UCSF Chimera.^25^ The ligand information was converted to the parameter format that is compatible with the Rosetta generic potential (*GenFF*.^24^). The initial position of the ligand to initiate docking was determined by superimposing the complex crystal structure on the sampled protein backbone. Protein–ligand docking was performed by allowing the side chains that are within 6A of the ligand to be flexible. The receptor models were optimized in advance using Rosetta FastRelax with high constraints on each backbone. We ran 20 parallel docking runs for each receptor model and ligand pair, and the combined results were analyzed, where the best scoring generated sample was compared to best scoring models of the training set, training crystals, and AlphaFold models.

## Supporting Information Available

Main findings, offering additional insights and analyses; figures illustrating the comparison of reconstruction performance between different VAE models, the relationship between latent dimension and distance RMSD error, and the impact of the number of samples generated in the latent space on accuracy; distribution analyses of C-alpha coordinate RMSDs, and detailed comparison tables provide further context (PDF)
