# Supplementary material for: Protein Ensemble Generation Through Variational Autoencoder Latent Space Sampling
Source: J Chem Theory Comput. 2024 Mar 28;20(7):2689–95. doi: 10.1021/acs.jctc.3c01057 (PMC11008089; doi:10.1021/acs.jctc.3c01057)
Supplement: Supplementary file 1 — ct3c01057_si_001.pdf [file ct3c01057_si_001.pdf]

Supplementary Information

Protein Ensemble Generation through Variational Autoencoder Latent Space Sampling

Sanaa Mansoor[1,2,3*], Minkyung Baek[1,2,4], Hahnbeom Park[1,2,5], Gyu Rie Lee[1,2], David Baker[1,2,6]

1. Department of Biochemistry, University of Washington, Seattle, WA 98195, USA.
2. Institute for Protein Design, University of Washington, Seattle, WA 98195, USA.
3. Molecular Engineering Graduate Program, University of Washington, WA 98195, USA.
4. School of Biological Sciences, Seoul National University, Seoul, 08826, Republic of Korea.
5. Brain Science Institute, Korea Institute of Science and Technology, Seoul, 02792, Republic of Korea
6. Howard Hughes Medical Institute, University of Washington, Seattle, WA 98195, USA.

* Corresponding author: sanaamansoor@google.com

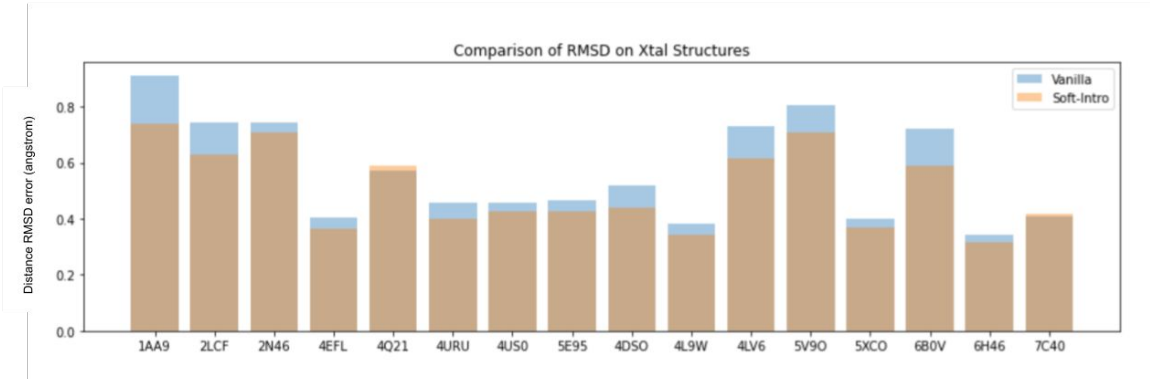

Figure S1. Comparison of reconstruction performance between vanilla VAE and soft-introspective VAE. Distance RMSD error (angstroms) comparison of reconstruction of a different set of K-Ras training crystals from similarly trained vanilla VAE and soft-introspective VAE.

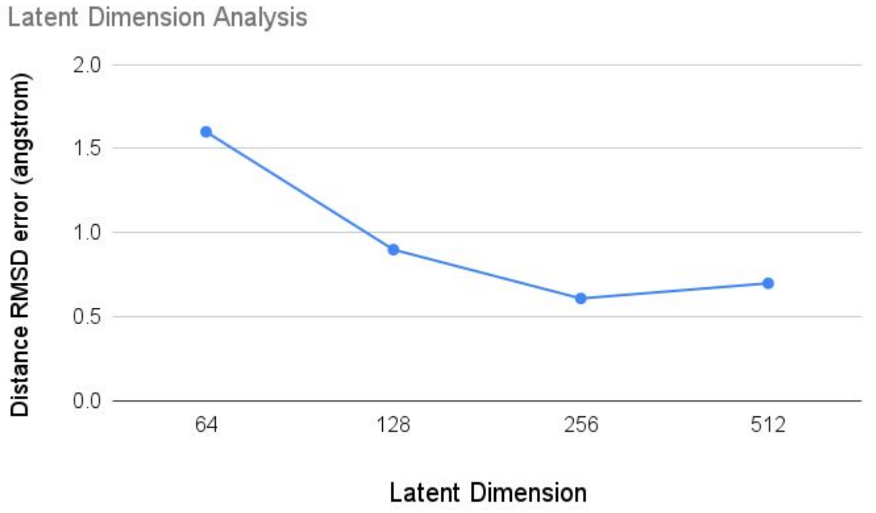

Figure S2. Graph illustrating the relationship between latent dimension and distance RMSD error. Increasing the dimension (x-axis) initially leads to a significant decrease in mean distance RMSD calculated over training data (y-axis), indicating improved data representation. However, the graph reaches an elbow point (256 dimensions) where further dimension expansion yields diminishing returns, plateauing the distance RMSD reduction.

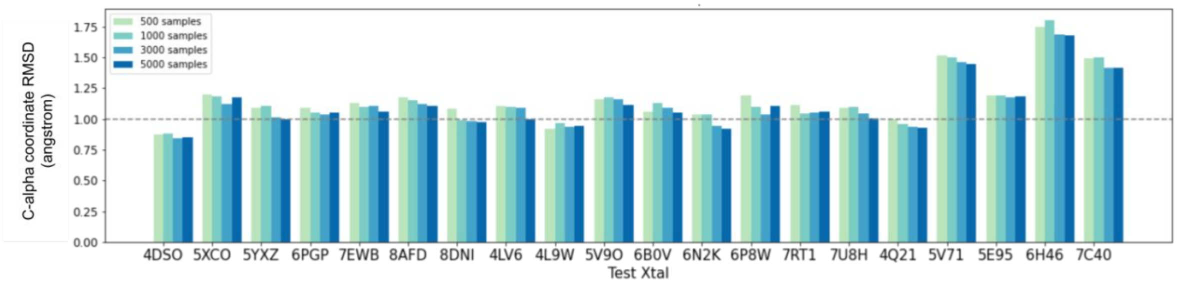

Figure S3. Relationship between increasing number of samples generated in latent space and closest C-alpha coordinate RMSD to target. Each target is associated with a different number of samples generated in the latent space, and the corresponding closest C-alpha Coordinate RMSD to the target crystal is plotted. More samples result in lower RMSD until a threshold is reached, indicating improved accuracy.

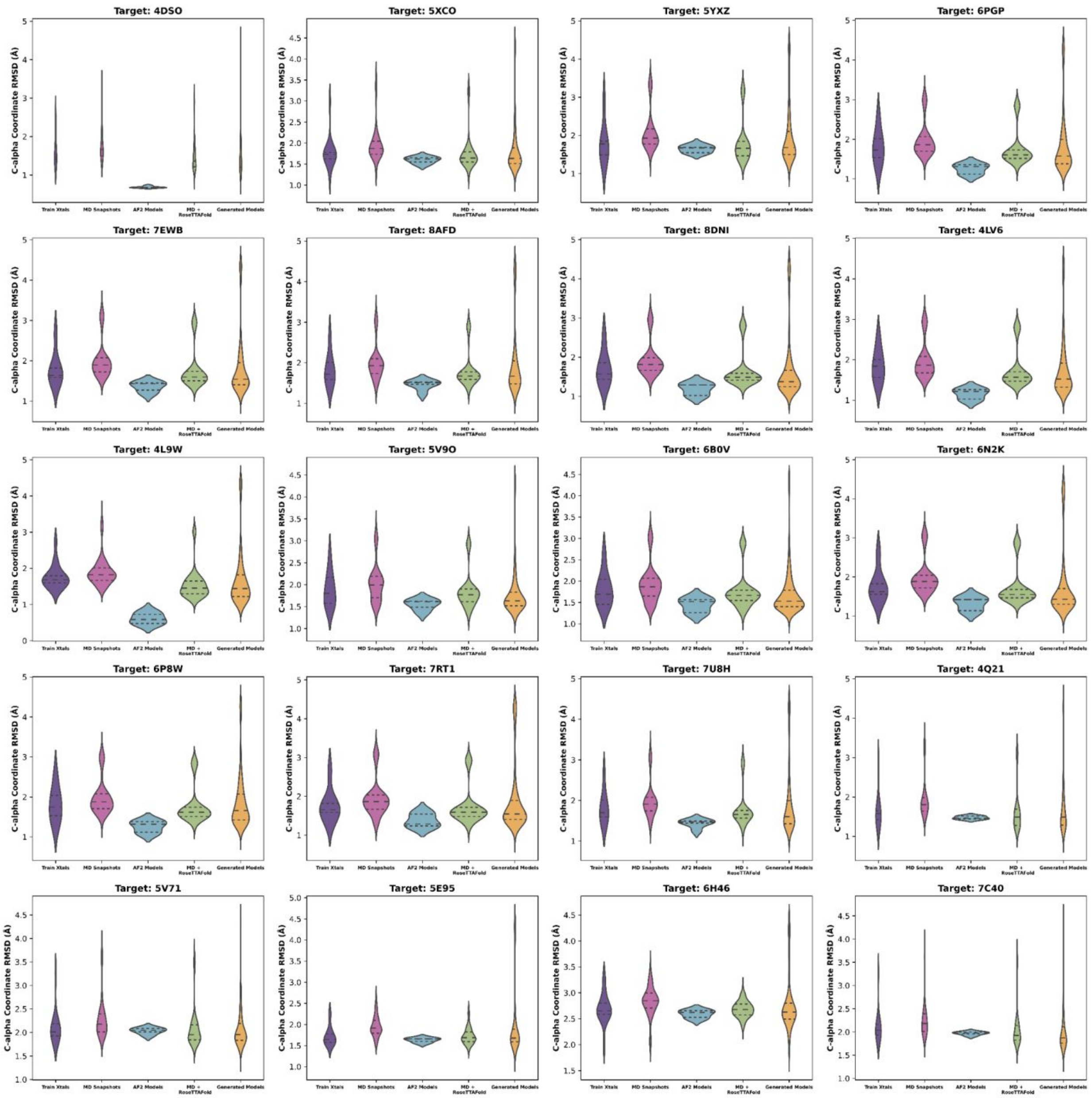

Figure S4: Distribution Analysis of C-alpha Coordinate RMSDs. Violin plots illustrate the distribution of C-alpha coordinate RMSDs for distinct categories. The analyzed categories include train crystals, MD snapshots, AF2 models, MD + RoseTTAFold models and generated models. Each plot represents the C-alpha coordinate RMSD distribution for a specific target, providing insights into the structural variations within each category.

Table S1. Reconstruction Accuracy Comparison. This table compares AF2 predictions and the trained VAE model reconstructions of the target crystal. 13 out of 20 targets achieved sub-angstrom accuracy, contrasting with only 2 out of 20 AF2 predictions.

Target Name	AF2 Model	VAE Reconstruction
4DSO	0.6742575085	0.8071033876
5XCO	1.619361527	1.164887154
5YXZ	1.68678524	0.8444671706
6PGP	1.355076683	0.9468465377
7EWB	1.432819558	0.9196066331
8AFD	1.521645539	1.068183021
8DNI	1.289528774	0.746212664
4LV6	1.260482257	0.8771103494
4L9W	0.4784715956	0.7701738878
5V9O	1.645989744	1.08979633
6B0V	1.563902278	0.9067739276
6N2K	1.418004407	0.9846375913
6P8W	1.38165785	0.8944044618
7RT1	1.240424989	0.899485806
7U8H	1.489494233	0.9812120675
4Q21	1.517058615	0.8033942649
5V71	2.095615225	1.461646455
5E95	1.661072709	1.16061586
6H46	2.620930292	1.723214959
7C40	2.0038039	1.315308216

Table S2: Minimum C-alpha RMSD and Standard Deviation per Target in Each Category.

This table displays the minimum C-alpha RMSDs and their standard deviations for each target across different structural categories, including training set crystals, MD snapshots, AF2 models, MD + RoseTTAFold models, and generated models. The last row of this table provides the results of the Mann-Whitney U test comparing the coordinate RMSD distributions between each category and the generated models. All p-values obtained from the Mann-Whitney U test are statistically significant, indicating significant differences between the generated models and the other structural categories.

	Minimum Coordinate RMSD +/ std for category				
Target	Train Xtals	MD Snapshots	AF2 models	MD + RoseTTAFold	Generated Models
4DSO	1.218 +/- 0.401	1.159 +/- 0.425	0.669 +/- 0.024	0.843 +/- 0.430	0.848 +/- 0.813
5XCO	1.202 +/- 0.362	1.197 +/- 0.422	1.481 +/- 0.078	1.118 +/- 0.414	1.114 +/- 0.623
5YXZ	1.202 +/- 0.545	1.201 +/- 0.541	1.532 +/- 0.099	1.137 +/- 0.580	0.989 +/- 0.757
6PGP	1.218 +/- 0.443	1.205 +/- 0.446	1.103 +/- 0.129	1.178 +/- 0.452	1.029 +/- 0.785
7EWB	1.407 +/- 0.418	1.393 +/- 0.471	1.173 +/- 0.128	1.235 +/- 0.487	1.021 +/- 0.795
8AFD	1.422 +/- 0.402	1.214 +/- 0.440	1.237 +/- 0.124	1.194 +/- 0.425	1.109 +/- 0.862
8DNI	1.247 +/- 0.432	1.240 +/- 0.448	1.019 +/- 0.154	1.170 +/- 0.473	0.895 +/- 0.806
4LV6	1.388 +/- 0.413	1.323 +/- 0.463	0.989 +/- 0.134	1.272 +/- 0.474	0.975 +/- 0.733
4L9W	1.388 +/- 0.327	1.260 +/- 0.388	0.448 +/- 0.158	0.963 +/- 0.410	0.852 +/- 0.799
5V9O	1.478 +/- 0.390	1.272 +/- 0.444	1.353 +/- 0.124	1.157 +/- 0.415	1.123 +/- 0.535
6B0V	1.410 +/- 0.395	1.187 +/- 0.453	1.252 +/- 0.165	1.066 +/- 0.427	0.993 +/- 0.499
6N2K	1.360 +/- 0.413	1.339 +/- 0.457	1.111 +/- 0.172	1.187 +/- 0.477	0.911 +/- 0.838
6P8W	1.221 +/- 0.444	1.210 +/- 0.446	1.083 +/- 0.147	1.177 +/- 0.450	0.980 +/- 0.714
7RT1	1.270 +/- 0.418	1.091 +/- 0.478	1.215 +/- 0.177	1.044 +/- 0.468	0.917 +/- 0.861
7U8H	1.402 +/- 0.408	1.161 +/- 0.448	1.245 +/- 0.107	1.094 +/- 0.446	1.062 +/- 0.819
4Q21	1.270 +/- 0.470	1.256 +/- 0.508	1.423 +/- 0.043	0.992 +/- 0.558	0.929 +/- 0.802
5V71	1.810 +/- 0.355	1.700 +/- 0.423	1.928 +/- 0.067	1.560 +/- 0.418	1.388 +/- 0.525
5E95	1.467 +/- 0.225	1.415 +/- 0.248	1.551 +/- 0.055	1.297 +/- 0.199	1.220 +/- 0.804
6H46	1.958 +/- 0.286	1.808 +/- 0.285	2.488 +/- 0.078	2.190 +/- 0.161	1.684 +/- 0.494

7C40	1.833 +/- 0.353	1.673 +/- 0.427	1.910 +/- 0.038	1.542 +/- 0.427	1.391 +/- 0.583
P-value	0.00011	0.00080	4.78E-11	0.00032	N/A

Table S3. Minimum C-alpha RMSD and Standard Deviation per Target in Each Category over Cryptic Pocket Residues.

This table displays the minimum C-alpha RMSDs and their standard deviations for each target across different structural categories calculated only over the cryptic pocket residues, including training set crystals, MD snapshots, AF2 models, MD + RoseTTAFold models, and generated models.

	Minimum Coordinate RMSD +/ std for category over cryptic pocket residues				
Target	Train Xtals	MD Snapshots	AF2 models	MD + RoseTTAFold	Generated Models
4DSO	0.275 +/- 0.120	0.258 +/- 0.237	0.199 +/- 0.034	0.239 +/- 0.151	0.261 +/- 1.441
5XCO	1.809 +/- 0.285	1.653 +/- 0.372	2.199 +/- 0.080	1.456 +/- 0.329	1.394 +/- 0.647
5YXZ	1.271 +/- 0.932	1.199 +/- 0.863	1.976 +/- 0.163	1.173 +/- 0.851	1.101 +/- 1.464
6PGP	1.914 +/- 0.497	1.727 +/- 0.454	1.713 +/- 0.283	1.641 +/- 0.487	1.302 +/- 0.843
7EWB	1.812 +/- 0.435	1.463 +/- 0.440	1.975 +/- 0.262	1.521 +/- 0.326	1.504 +/- 0.633
8AFD	1.969 +/- 0.422	1.307 +/- 0.488	1.902 +/- 0.292	1.365 +/- 0.356	1.331 +/- 0.819
8DNI	1.970 +/- 0.564	1.784 +/- 0.548	1.698 +/- 0.350	1.759 +/- 0.555	1.235 +/- 0.895
4LV6	2.177 +/- 0.647	1.888 +/- 0.710	1.664 +/- 0.308	1.741 +/- 0.747	1.348 +/- 1.099
4L9W	1.722 +/- 0.606	1.373 +/- 0.643	0.355 +/- 0.239	1.170 +/- 0.738	1.117 +/- 2.168
5V9O	2.191 +/- 0.519	1.639 +/- 0.559	2.307 +/- 0.278	1.609 +/- 0.498	1.445 +/- 0.535
6B0V	2.077 +/- 0.476	1.438 +/- 0.567	1.980 +/- 0.389	1.309 +/- 0.535	1.371 +/- 0.496
6N2K	2.145 +/- 0.414	1.789 +/- 0.430	1.836 +/- 0.378	1.828 +/- 0.447	1.419 +/- 0.789
6P8W	1.972 +/- 0.514	1.787 +/- 0.471	1.736 +/- 0.338	1.718 +/- 0.505	1.366 +/- 0.920
7RT1	1.957 +/- 0.392	1.271 +/- 0.476	1.906 +/- 0.384	1.541 +/- 0.325	1.309 +/- 0.540
7U8H	2.263 +/- 0.439	1.303 +/- 0.457	2.142 +/- 0.232	1.316 +/- 0.362	1.455 +/- 0.502
4Q21	0.348 +/- 1.030	0.334 +/- 0.824	1.167 +/- 0.045	0.400 +/- 0.913	0.529 +/- 1.980
5V71	1.589 +/- 0.557	1.392 +/- 0.593	2.241 +/- 0.083	1.440 +/- 0.584	1.513 +/- 0.946
5E95	0.288 +/- 0.066	0.294 +/- 0.265	0.283 +/- 0.009	0.314 +/- 0.179	0.336 +/- 0.910
6H46	0.989 +/- 0.334	0.808 +/- 0.362	1.611 +/- 0.009	0.983 +/- 0.216	0.782 +/- 1.517
7C40	0.134 +/- 0.066	0.126 +/- 0.309	0.182 +/- 0.013	0.151 +/- 0.220	0.181 +/- 0.440

Table S4. Lowest C-alpha Coordinate RMSD over cryptic pocket residues of docked structures.

	Minimum C-alpha Coordinate RMSD +/ std for category over cryptic pocket residues			
Target Name	Train Xtal	MD Snapshot	AF2 Model	Generated Model
4LV6	3.599	1.665	1.448	1.151
5YXZ	4.055	1.043	1.876	1.13
5V71	1.407	1.058	1.863	0.972
6PGP	3.641	1.671	1.667	1.185
6N2K	3.826	1.657	1.803	1,355

Table S5. Lowest RMSD over ligand atoms (ligand RMSD).

	Minimum Coordinate RMSD +/ std for category over ligand atoms			
Target Name	Train Xtal	MD Snapshot	AF2 Model	Generated Model
4LV6	5.222	1.513	4.681	1.3
5YXZ	2.821	1.079	3.569	0.765
5V71	0.863	0.941	4.102	0.889
6PGP	9.479	2.028	3.678	1.363
6N2K	9.108	1.657	5.007	1.585

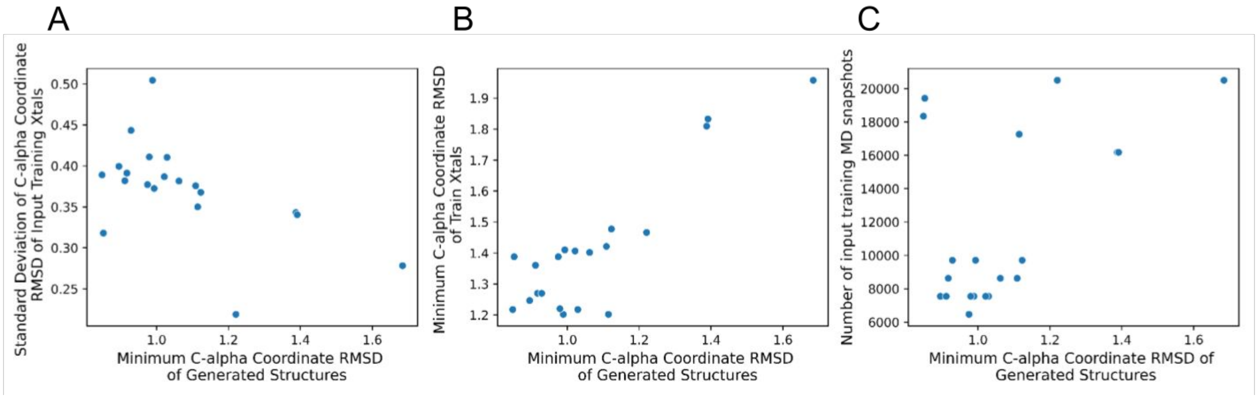

Figure S5. Relationship between input quality and generated structure quality. A) Scatterplot depicting how the quality of generated structures correlates with the variability in RMSDs of training crystals to the target. Each x-axis point represents the RMSD of the closest generated structure, while the y-axis shows the standard deviation in RMSDs of training crystals. B) Scatterplot illustrating the relationship between generated structure quality and input training crystal quality. Each x-axis point represents the RMSD of the closest generated structure, and the y-axis shows the RMSD of the closest training crystal used for MD simulations. C) Scatterplot highlighting the relationship between generated structure quality and the number of input training MD snapshots. Each x-axis point represents the RMSD of the closest generated structure, while the y-axis shows the number of input MD snapshots used to train the VAE model for each target. Adding more than 6000 simulation structures showed diminishing returns, suggesting limited improvement beyond this point. Our model, with 1.5 million parameters, minimizes overfitting but raises concerns about underfitting due to dataset size.
